# Bridging gaps in community cancer care: exploring Traditional Health Practitioners’ perceptions of common adult cancer and their role in raising community awareness and early detection in Soweto, South Africa

**DOI:** 10.1080/16549716.2026.2685414

**Published:** 2026-07-13

**Authors:** Ngcwalisa Amanda Jama, Maureen Joffe, Keletso Mmoledi, Seemela D. Malope, Gugulethu Tshabalala, Charmaine Blanchard, Elizabeth F. Msoka, Shane A. Norris

**Affiliations:** a SAMRC/Wits Aging African Adults Research Unit, Faculty of Health Sciences, University of the Witwatersrand, Johannesburg, South Africa; b Network for Oncology Research in Africa (NORA), Global Health Working Group, Martin-Luther-University, Halle-Wittenberg, Germany; c Perinatal HIV Research Unit (PHRU), University of the Witwatersrand, Johannesburg, South Africa; d African Social Sciences Unit of Research and Evaluation (ASSURE), Wits Health Consortium, University of the Witwatersrand, Johannesburg, South Africa; e Global and Planetary Health Working Group, Martin-Luther-University, Halle-Wittenberg, Germany; f Kilimanjaro Clinical Research Institute, Kilimanjaro Christian Medical Centre, KCMC University, Moshi, Tanzania; g School of Human Development and Health, University of Southampton, Southampton, UK

**Keywords:** Traditional healers, adult cancer, knowledge, traditional-biomedical collaboration, sub-Saharan Africa

## Abstract

**Background:**

Despite the rising cancer burden in Soweto, South Africa and the recognized role of Traditional Health Practitioners (THPs) in the pluralistic health system, their role in cancer care remains underexplored. This study provides novel insights into THPs’ understanding of cancer and their potential contribution to community awareness and early detection within complex sociocultural and health system contexts.

**Objective:**

To explore THPs perceptions of cancer and the factors influencing their role in community cancer awareness and early detection in Soweto.

**Methods:**

An exploratory qualitative design was adopted. Purposive and snowball sampling were used to recruit 13 THPs in Soweto. Each participated in two in-depth interviews. Data were transcribed, coded, and thematically analyzed using Dedoose.

**Results:**

Seven of the 13 THPs had encountered clients with cancer-related symptoms. The THPs described navigating cancer care with mixed certainty, drawing on experiential, spiritual, and limited biomedical knowledge. They often referred clients to biomedical facilities when symptoms appeared serious or unclear. Participants saw themselves as accessible community figures, offering psychosocial support and helping patients navigate the health system. While motivated to contribute to cancer awareness and early detection, they reported challenges related to limited recognition and legitimacy within the formal health sector.

**Conclusion:**

Traditional Health Practitioners play an informal but important intermediary role in community cancer care in Soweto. Their contributions are shaped by knowledge gaps, social dynamics, and systemic barriers. Strengthening their role in early detection requires interventions that improve training, build trust in biomedical services, and support integration within the health system.

## Paper context

- **Main findings:** This study provides novel insights into the underexamined role of THPs in community-based cancer care within South Africa’s pluralistic health system.
- **Added knowledge:** The study shows that THPs are motivated to promote cancer awareness and early detection and serve as accessible community intermediaries, but their capacity is limited by insufficient training, systemic barriers, and lack of formal recognition.
- **Global health impact for policy and action:** The findings highlight the potential of strengthened collaboration between traditional and biomedical systems to support earlier cancer detection and inform integration strategies in pluralistic health systems.

## Background

South Africa faces a rapidly increasing cancer burden, with cancer being responsible for approximately 10% of all deaths and its incidence projected to rise substantially in the coming decades [1,2]. Many cancers in the country are diagnosed at advanced stages, resulting in markedly poorer survival outcomes [3,4]. Delayed cancer diagnosis in many resource-constrained settings, such as South Africa has been strongly linked to limited cancer awareness, cultural and socioeconomic barriers, screening underuse, fragmented pathways to care, and weak engagement with community-level healthcare actors [5–8]. Consequently, national and global cancer strategies increasingly emphasize awareness and early detection as critical approaches for improving timely diagnosis and cancer outcomes [9,10].

For South Africa, addressing persistent gaps in cancer awareness and seeking help early may require culturally responsive, community-level approaches that engage trusted local healthcare actors. In this context, Traditional Health Practitioners (THPs), including diviners, herbalists, traditional birth attendants, and traditional surgeons as formally recognized under the THPs Act, No. 22 of 2007 have a key role to play [11]. Traditional Health Practitioners are highly trusted and accessible community-based providers who are frequently consulted for various health concerns, including cancer [12–16]. Specifically, South Africa’s National Cancer Strategic Framework acknowledges the potential contribution of THPs to prevention, screening, early detection, survivorship, and palliative care [10].

Despite increasing policy recognition of THPs and their potential contributions to prevention and early detection, collaboration between THPs and biomedical health practitioners (BHPs) remains largely informal and inconsistently implemented within practice settings [17]. Additionally, little is known about how THPs understand cancer, perceive their roles in cancer awareness and referral, and navigate interactions with biomedical services, particularly in urban South African settings. The only recent study examining collaboration with oncology services has identified barriers related to limited professional relationships, communication challenges, and lack of patient inclusion in treatment decisions [18].

Important questions, therefore, remain regarding how THPs conceptualize cancer, perceive their role in cancer care, and engage with biomedical services. This study explored THPs’ perceptions of cancer and the factors influencing their role in community cancer awareness and early detection in Soweto, South Africa. Understanding THPs’ perspectives may help in informing culturally appropriate cancer awareness interventions, strengthening referral pathways between traditional and biomedical systems, and guiding future policy, practice, and research on collaboration within pluralistic health systems in South Africa and similar settings.

### Conceptual framework

**Figure 1.**
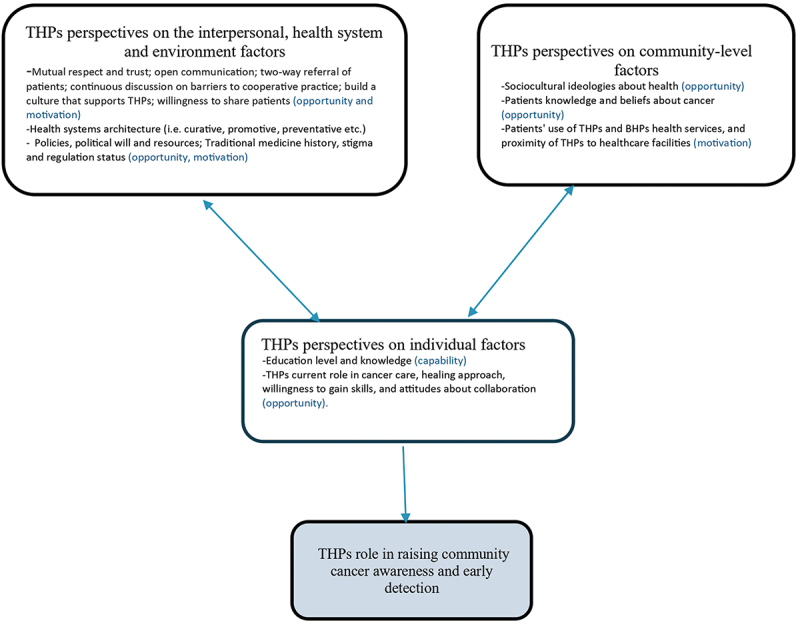
Conceptual framework illustrating how the SEM [19], the COM-B [20] and the THP-BHP cooperative model [18] interact to influence THPs’ involvement in cancer interventions.

This study was informed by the Social Ecological Model (SEM), the Capability, Opportunity, Motivation-Behavior (COM-B) model, and Nkosi and Sibiya’s THP-BHP cooperative framework [17–19], as illustrated in Figure 1. The SEM guided the exploration of how individuals, communities, and the health system influence the roles of THPs in cancer awareness and early detection. The COM-B model informed the interpretation of factors shaping THPs’ capabilities, opportunities, and motivations, while the cooperative framework contextualized collaboration between traditional practitioners and BHPs.

## Methods

### Study design and setting

We conducted an exploratory qualitative study in Soweto, a township in southwest Johannesburg, South Africa. This design enabled the investigation of an underexplored phenomenon and the generation of insights relevant to urban community engagement in cancer research [21]. An interpretive approach guided the study, focusing on the social, cultural, and contextual factors that shape the perspectives and practices of THPs.

### Participants and recruitment

Purposive and snowball sampling were used to recruit THPs practicing in Soweto. Eligible participants were aged 18 years or older, resided in and actively practiced in Soweto, and had a minimum of 1 year’s experience working with clients as THPs. Participants could be either registered or unregistered with professional bodies and fall into any THP category. Recruitment began with three purposively selected participants who were known within local THP networks and who met the study inclusion criteria. These participants represented different traditional healing practices and were selected to facilitate initial access to the broader THP community in Soweto. Recruitment was subsequently expanded through participant referrals using snowball sampling. Recruitment continued until 13 THPs were enrolled and interviewed. At this point, the research team determined that sufficient information power had been reached, as subsequent interviews after the 10th participant no longer generated substantially new insights or thematic dimensions relevant to the study objectives [22].

### Data collection

Data were collected from April to June in 2025 through in-depth interviews. Two experienced qualitative researchers (NAJ and KM) conducted the interviews in *IsiZulu, IsiXhosa*, or *SeTswana*, depending on participant preference. Each participant completed two interviews approximately 2–4 weeks apart, resulting in a total of 26 in-depth interviews. Two interview guides were accordingly developed to address study objectives, informed by the SEM, the COM-B model, and the THP-BHP cooperative framework (Supplementary file 1). The first guide helped us to explore THPs’ practices, experiences with clients presenting with cancer-related symptoms, perceptions of cancer, awareness and early detection. The second involved summary validation and further discussion of THPs’ potential roles in cancer awareness and early detection and supporting a collaborative approach. The interview process was iterative, allowing emerging issues from earlier interviews to inform subsequent discussions and probing. The interval between interviews additionally allowed participants time to reflect on the research phenomenon and facilitated the development of rapport and trust between participants and researchers, particularly given mistrust toward researchers within some THP communities. Interviews took place at a community center in Soweto or at the participant’s home, depending on their preference. The interviews were audio-recorded and lasted between 30 and 120 min.

### Data analysis

The transcripts were translated into English and analyzed using Braun and Clarke’s Reflexive Thematic Analysis [23]. Reflexive Thematic Analysis emphasizes researcher reflexivity, interpretation, and active engagement in knowledge production rather than assuming that themes simply emerge from the data. In practice, NAJ conducted the initial familiarization and inductive coding of transcripts and notes in Dedoose (v10.0.34). The coding process combined inductive and deductive approaches [24] whereby initial codes were generated from participants’ accounts and emerging patterns in the data, while the study’s conceptual frameworks and interview guides informed code development and interpretation. Moreover, KM, GT, and DM contributed as co-coders and refined codes and themes through discussion. Previously coded transcripts were reviewed to ensure consistency and codes were merged, expanded, or renamed as the analysis progressed. A final analytical report with the agreed themes was produced by the research team. This process was repeated for the second-phase interviews.

### Trustworthiness and rigor

Key epistemological standards were maintained to ensure the rigor and trustworthiness of the study [25]. Credibility was enhanced through the involvement of two interviewers during data collection, member checking during the second interviews, and independent, double coding of all transcripts by members of the research team to support analytic rigor. Consistent with Reflexive Thematic Analysis, interrater reliability was not calculated, as the aim was not to achieve statistical agreement but to deepen reflexive and interpretive engagement with the data. Coding differences were discussed collaboratively, and codes and themes were refined through iterative team discussions. Confirmability was supported through reflexive journaling and maintaining an audit trail documenting methodological and analytic decisions throughout the study, including recruitment processes, interview reflections, code development, and theme refinement. Dependability was ensured through rigorous coding procedures and documentation of the analytic decisions. Transferability was strengthened through detailed reporting of the context and participant characteristics.

#### Reflexivity

Researchers’ positionalities shaped the interpretation of the data. NAJ, a PhD social scientist, has engaged in the research on THPs’ integration for the past seven years and led the fieldwork and analysis. KM, a palliative care nurse and public health researcher, contributed to the clinical expertise and familiarity with the local context. Other team members with backgrounds in biosocial cancer research but limited field involvement provided critical external perspectives during the analysis.

## Results

### Participant characteristics and exposure to client cancer care

Thirteen THPs practicing in Soweto participated, and their profiles are detailed in Table 1. Experience ranged from 3 to 49 years, with some initiating in adolescence. Most identified as *Izangoma* (diviners), while others were *Izinyanga* (herbalists), faith healers, or held hybrid roles. Eight were affiliated with healer associations and had training in HIV, hypertension, tuberculosis, diabetes, and mental health, but none had formal cancer training. Seven THPs reported encounters with clients presenting cancer-related symptoms or diagnoses (e.g. breast, prostate, cervical, skin, leukemia, blood, and bone cancers), while six THPs reported none.

**Table 1.** Characteristics of THPs (*N* = 13).

	Category	N (%)
Type of practitioner	Sangoma/diviner only	7 (53.8)
	Inyanga/herbalist only	1 (7.7)
	Faith healer only	1 (7.7)
	Sangoma, Inyanga, and Faith healer	2 (15.4)
	Inyanga and Faith healer	2 (15.4)
Years since initiation	1–10 years	5 (38.5)
	11–20 years	3 (23.1)
	21–30 years	2 (15.4)
	More than 30 years	3 (23.1)
Registered/affiliated with professional body	Yes	8 (61.5)
	No	5 (38.5)
Experience with cancer-related cases	Yes	7 (53.8)
	No	6 (46.2)
Attendance of NDoH/NGO workshops	HIV/AIDS	9 (69.2)
	Hypertension	8 (61.5)
	Tuberculosis	2 (15.4)
	Diabetes	2 (15.4)
	Mental health	1 (7.7)
	Cancer	1 (7.7)

### Overview of themes

Our analysis revealed four interrelated themes that illuminate how THPs position themselves in relation to cancer care, operating at individual, community, and institutional/health system levels. The themes and accompanying subthemes are organized in Table 2.

**Table 2.** Study themes and subthemes organized according to the SEM levels.

Theme	SEM level	Subthemes
1. Navigating cancer with uneven certainty	*Individual*	1.1 Limited formal cancer knowledge and experiential learning 1.2 Uncertainty around symptoms and post-treatment effects 1.3 Spiritual diagnosis 1.4 Between referral and co-treatment
2. Community perceptions of cancer and patterns of help-seeking	*Community/interpersonal*	2.1 Trust in THPs 2.2 Gendered healthcare-seeking 2.3 Fear, witchcraft, and stigma
3. Motivation and concerns regarding engagement		3.1 Willingness to support cancer awareness initiatives and desire for training 3.2 Concerns about exploitation of indigenous knowledge
4. Negotiations of legitimacy within the formal health system	*Health system*	4.1 Lack of recognition and exclusion from consultations 4.2 Mistrust between THPs and BHPs 4.3 Calls for formal collaboration

## Navigating cancer with uneven certainty

### Limited formal cancer knowledge and experiential learning

At the individual level, THPs indicated varying degrees of cancer knowledge, limited formal training, and uncertainty in recognizing and managing cancer-related symptoms. The prevailing perception of cancer among most was that it was a fatal disease, complex to discern, particularly through THPs’ diagnostic methods, and uncurable. Some associated the cause of cancer with hereditary factors or lifestyle influences, such as diet, smoking, and alcohol use, while others linked it to broader bodily or spiritual disturbances. They expressed that cancer can be managed or treated if caught early, with some THPs citing the survival stories of their past clients as evidence of successful treatment. The THPs were hopeful about prevention, with some suggesting that cancer could be avoided through lifestyle changes, such as regular check-ups, healthy eating, and avoidance of alcohol and smoking.

Some participants reported on the knowledge gap, calling for more opportunities to learn about cancer, as illustrated below:

I don’t think we are well educated about cancer symptoms because as traditional healers, we were asked by our government to attend uhm … our workshops where they certified us. So, mostly it was HIV and AIDS … It was hypertension, diabetes … but not cancer. Cancer was not there. But the first question I would ask you is, What are cancer symptoms? You know, maybe I’m aware but I’m not sure if it is cancer. But yes, because now we read, we’ve never really had a proper session where we are taught to say, ‘Guys, watch out for these and these symptoms; this is cancer.’ But again, because of us being young healers, now we are aware of cancer. (THP 11, IDI 1)

Ever since I have been in this field, it is my first time to meet people [researchers] who are coming to engage me about cancer. I do not remember attending a workshop or a training for cancer. (THP 06, IDI 1)

### Uncertainty

Uncertainty often arose when clients presented with complex post cancer-treatment side effects. Some revealed how this information gap inhibits accurate interpretation of clients’ symptoms, calling for NDoH to educate THPs on cancer:

I had a client that was complaining of pain. But now the confusion because remember I said there isn’t any … formal knowledge on our side when it comes to cancer, for example, or chemo and maybe if there is a removal of a certain organ like maybe the breast, the womb. So, to us, that is entirely new. So I had to do research on my own because I’m like, she doesn’t have a womb. So where’s the pain coming from? And then I did an herbal tonic to detox. So it was quite confusing … So, we are appealing to the government to organize those workshops for us on signs and symptoms of cancer … post-treatment organ removal … . Basic things so that we are also aware. (THP 11, IDI 2)

### Spiritual diagnosis of cancer

Traditional diagnostic practices, such as divination and ancestral guidance, were described as offering insight into illness but not necessarily biomedical specificity. Participants explained that spiritual guidance might reveal that ‘the blood has a problem’ but not identify the disease as cancer. In these situations, referral to a clinic was considered necessary to obtain biomedical confirmation:

As a THP, and pertaining to cancer, you can spiritually sense as per what your ancestors are telling you that this person is sick. They will signal that the sickness is in the blood, but they will not tell you that it is cancer or diabetes. No! But they will tell you that the blood of this person is the one that has a problem. Remember that I do not have Western equipment to check on people, but I can take you to the clinic or I can refer you to the clinic. And then after going to the clinic, that’s when I can help you because I would have now heard what the Western doctor said. (THP 06, IDI 1)

### Between referral and co-treatment

Despite these uncertainties, many THPs described playing a supportive role for clients who were faced with possible cancer diagnoses. These roles included counseling clients, dispelling beliefs linking cancer to witchcraft, encouraging treatment adherence, and supporting emotional wellbeing.

Participants also expressed uncertainty about the boundaries of their professional roles when addressing illnesses such as cancer, particularly in situations involving referral or co-treatment with biomedical providers. Theyoften described referring clients to biomedical facilities when symptoms suggested serious illness or when diagnostic procedures, such as imaging or laboratory tests, were required. In some cases, THPs accompanied clients to clinics to ensure that they accessed the appropriate care:

So, they [the family] told me that this patient was told in [province X] that she has cancer and told the boyfriend that she left work because they were bewitching her at work. But in my assessment, I noticed … this must be cancer. I noticed she was reluctant to go to the hospital; then for the first two weeks, I gave her my herbal tonic. It was supposed to drain water and redness. I also wanted to confirm my suspicions. Because of witchcraft, she didn’t want me to check. She just said, ‘This is my problem, and I need this [herb].’ … But, when I checked, I could not see witchcraft. I then said, ‘What I am seeing here is medical … .’ But I further said, ‘I am not in a position to give you something that can deal with your condition right now.’ That’s when she agreed to go to the clinic with me. (THP 05, IDI 2)

Some participants described providing herbal remedies or spiritual support in addition to biomedical care, particularly when clients returned during or after hospital treatment. In these cases, traditional care was framed as complementary, addressing the spiritual or emotional aspects of illness rather than replacing biomedical treatment:

I work a lot with Western doctors who know that my herbs have been publicly endorsed even though they’ve never tested at the laboratory per se. But, uh, they’ll refer and say, ‘[G]o and see this traditional healer.’ So, some of my clients, I get them in that manner. And then later on when they go and test, they do find that that cancer has halted its spread. (THP 05, IDI 1)

## Community perceptions of cancer, patterns of help-seeking, gendered access to cancer

### Trust in THPs

At the community level, participants highlighted how stigma, fear, cultural beliefs, and gendered help-seeking patterns shaped community engagement with cancer care. In examining THPs’ perceptions of their role in cancer care within Soweto, participants indicated that cancer remains a relatively silent topic in their communities. In contrast to HIV, which has been the focus of sustained awareness campaigns, cancer has rarely been publicly discussed. As a result, participants perceived cancer as less understood and less normalized in everyday discourse, with some believing that interventions aimed at cancer awareness would enable such conversations to take place:

Cancer is not a common subject in our community. I think it is a less talkative thing. They can only speak about it like when someone can say, ‘I have this friend of mine that I know’; then that’s when the topic is going to start. But other than that, I don’t think people speak about cancer here in my community. (THP 02, IDI 1)

Within this context, THPs described themselves as accessible and trusted providers whose services aligned with clients’ cultural beliefs and health-seeking practices.

### Gendered healthcare-seeking

The participants also highlighted gendered patterns in healthcare access. Several noted that men were less likely to attend clinics and often delayed seeking biomedical care, particularly for conditions involving sensitive symptoms, such as urinary difficulties. THPs 03 and 05 specifically noted that the unwelcoming environment of clinics for men is one of the reasons they are frequently approached, particularly in cases involving prostate cancer symptoms:

Interviewer: And you said with those two cases, they were not diagnosed yet, but you could see it? Participant: It was prostate. This man did not like going to the clinic at all. So he went to the clinic because I took him; I accompanied him. And you know that for men and for different reasons, a clinic is very men unfriendly. Nurses are trying but they are not cracking it. They are not cracking it … . I think it is just in their [nurses] nature. (THP 05, IDI 1)

### Fear, stigma, and witchcraft

Participants also observed that clients often responded to cancer symptoms with fear, denial, or misconceptions about the disease. These perceptions are viewed as potential entry points for education and awareness as stated by one healer:

If a healer says, no guys, this is not witchcraft, and it is not [ilumbo], but it is cancer … that changes the whole diversion now [of how the community views cancer]. (THP 04, IDI 02)

However, some THPs cautioned that broader community engagement could be challenging due to mistrust of traditional practitioners. For this reason, many preferred to discuss cancer within the privacy of consultations rather than through public awareness activities, as reflected below:

In terms of that [THP involvement], I really don’t know because when it comes to the community, we are so different. So, our take depends on who is talking. For example, if you can say, ‘We have a traditional healer here who wants to teach the community about cancer, the community might say, ‘This one wants to manipulate witchcraft to enrich themselves by using us in the process.’ So, it is difficult. Yes, if we want to train other traditional healers, it will be easy but if we are to include the community at large, then it will be difficult. (THP 07, IDI 2)

## Motivation and concerns regarding engagement

### Willingness to support cancer awareness initiatives

Despite these challenges, the participants expressed a strong interest in contributing to cancer awareness and early detection initiatives. Many described a sense of responsibility for sharing health information within their communities, ‘*We’re more than willing. We’d love to sit and learn’* (THP 08, IDI 2).

Participants emphasized the need for structured training, educational materials, and opportunities to learn about cancer symptoms, treatment pathways, and referral processes:

I can say that cancer is more problematic than diabetes and HIV in our society. So if we can get support in terms of resources, we can use them and also have sessions with the community in the clinics. And we can, therefore, have the opportunity to encourage people to uptake screening. I am sure you can notice tents for HIV everywhere but where are those tents for cancer? This is what we are always complaining about … . It will be beneficial also to us … . Capacitate us. We want to actually professionalize our sector. (THP 05, IDI 2)

### Concerns about exploitation

However, this willingness was accompanied by concerns about exploitation and professional vulnerability. Some participants feared that sharing traditional knowledge, particularly herbal remedies, would lead to appropriation without formal acknowledgment. This came from some THPs who expressed that their remedies were effective in treating cancer symptoms; they perceived engagements with Western practitioners as a platform where this knowledge might be exploited:

We need knowledge so that we improve our health system, yeah, but then there’s also that fear of … I pass on the knowledge and I disappear [get killed] or exploited … something that I didn’t look at as a business, but here is somebody cashing in on it, yet I shared this information. (THP 08, IDI 1)

## Negotiations of legitimacy within the formal health sector, including experiences of exclusion

### Lack of recognition and exclusion from consultations

At the institutional level, the THPs described challenges related to legitimacy, professional recognition, and limited integration into formal healthcare systems. Although the participants described some ongoing interactions with the formal health system, particularly through referrals and accompanying clients to healthcare facilities, these interactions often highlighted structural and interpersonal barriers. One of these obstacles included lack of recognition of THP referral letters and sick notes. Some THPs reported encountering negative attitudes from healthcare providers when bringing clients to clinics. They were excluded from consultations or asked to wait outside, as illustrated in the account below:

There was this guy who came presenting with sores. And I told him, ‘Just go to the clinic because I cannot lie and say I will be able to treat you.’ I even accompanied him. And he took long before coming back to me. At first, they [the nurses] were like, ‘Why have you brought this person here [to the clinic] because we cannot hear from you … ?’ Some said, ‘You are crowding our space here as if this is your clinic.’ ‘Why do you have to accompany them here and not let them come on their own … ?’ I said to them, ‘[T]his person came to me as a traditional healer, and I noticed that I did not understand what their problem was; hence, we decided to come and consult you instead.’ I did that until they got used to it. (THP 10, IDI 2)

Participants expressed frustration that medical certificates issued within traditional practices were not recognized by formal employers, reinforcing perceptions of unequal professional acknowledgment:

Participant: Those who are registered with the clinic and GTFMP and THP have referral letters … . We also have sick notes. I can issue a sick note … It’s not acknowledged. If you would have a referral from the clinic or hospital and then they would read it, and then they would say, ‘Okay.’ But with our referral letters, they would say, ‘Where is this sangoma coming from anyway?’ (THP 11, IDI 2)

### Mistrust between THPs and BHPs

THPs reflected deeper concerns about unequal power relations and exclusion within existing models of engagement with the biomedical sector. While THPs expressed willingness to support referral and awareness efforts, they described experiences in which their involvement appeared instrumental and temporary, rather than collaborative or mutually beneficial:

[I]t seems like we are being used. It’s as if we are auxiliary workers who can just be used and after using us, then it is like … ‘[W]e are done with you.’ So, this is the main challenge that we had and that’s why they are fighting to say, give us that recognition. So, to them, it is like when they want to implement something, they want us to be part but at the end of the day, they leave us behind. So why not continue with us? Why not give us more information? … That’s why we feel like we are being used because we benefit nothing. (THP 02, IDI 2)

It should be that I get a follow-up to say, come and see your patient. The patient is now going for chemo … step by step, so that I can know or feel like I’m part of this whole thing. (THP 11, IDI 01)

Participants also highlighted structural barriers such as the absence of formal registration numbers or professional recognition, which influenced how biomedical practitioners viewed their work. As one participant explained, nurses and doctors questioned the safety of their treatment regimens by asking,

Whatever that you did, was it tested? Is it 100%? What if it is the one that caused damage? They’re having those kinds of attitudes towards us because we do not have a formal practice number. That’s why I say lack of recognition is the one that disadvantages us. (THP 03, IDI 2)

### Calls for formal collaboration

Beyond recognition by the healthcare system, the THPs expressed interest in structured regulations and government involvement. Some THPs pointed to the role of government structures in providing opportunities for dialogue and collaboration. They emphasized that regulations, education, and policies mandating Western-trained healers should be developed and implemented. One participant highlighted the need for an organized approach:

You see, so dialogue is needed. That is why you heard me say in the beginning ‘if the government’ because they [Western-trained healers] listen to the government. (THP 04, IDI 2)

Alongside calls for collaboration, THPs also cautioned that not all practitioners could be trusted, noting that some ‘take chances’ in their practice. As such, they emphasized that plans for collaboration with BHPs should take this concern into account.

… We also need to have a clear version of what we want to do because as traditional healers, there are those fly-by-night traditional healers who claim they can heal, but they can’t. So we need to reconcile ourselves first before we go out and say, ‘guys, we can help.’ Because we are not organized, and as traditional healers we are not united … . (THP 09, IDI 02)

## Discussion

This study explored how THPs in Soweto understand cancer, engage with biomedical services, and perceive their roles in community cancer awareness and early detection. Four key findings emerged. First, although THPs were frequently consulted by symptomatic clients, many reported limited formal cancer knowledge and uncertainty in recognizing cancer-related symptoms. Second, participants described themselves as trusted and accessible community practitioners who can facilitate help-seeking, particularly in contexts shaped by fear, stigma, denial, and gendered barriers to formal healthcare engagement. Third, most participants expressed strong motivation to support cancer awareness and referral if provided with appropriate training and support tools. Finally, their involvement was constrained by limited recognition within the formal health sector, including dismissive encounters, absent referral pathways, and regulatory ambiguity. Overall, our findings suggest that THPs occupy a negotiated position within South Africa’s pluralistic health system that may either support earlier engagement with cancer services or remain marginalized where structural barriers persist.

The key contribution of this study is a nuanced account of THPs’ cancer-related knowledge. Our findings suggest that cancer knowledge among THPs is not absent but is unevenly constructed through experiential practice, observation of illness trajectories, client interactions, and selective engagement with biomedical information. This challenges binary framings that position THPs as either knowledgeable collaborators or uninformed barriers to care. Importantly, this knowledge did not translate into categorical resistance to biomedical care. Instead, THPs in Soweto appeared embedded within informal referral and navigation processes across pluralistic care pathways where they acted as community educators who counseled clients, challenged misconceptions about cancer, and encouraged engagement with biomedical healthcare services.

The referral practices observed in this study suggest that many THPs recognize the limitations of traditional diagnostic approaches. As such, rather than functioning solely as alternative treatment providers, THPs may act as relational intermediaries who help clients navigate between traditional and biomedical systems. These findings highlight how cancer-care pathways in pluralistic settings are often negotiated across multiple healthcare systems rather than only following linear biomedical trajectories. In doing so, they challenge simplified assumptions that THPs uniformly contribute to delayed cancer diagnosis [13,26,27]. Cancer-control strategies that overlook THPs may, therefore, miss important community-level entry points for cancer awareness, referral, and early engagement with care [28].

From a COM-B model perspective, psychological capability is not only shaped by individual knowledge but also by shared informational frameworks that make health conditions understandable within communities. The social silence surrounding cancer suggests that awareness deficits are not simply informational gaps but also reflect limited collective frameworks through which communities can recognize, discuss, and respond to cancer symptoms. In such contexts, symptoms may be normalized, hidden, spiritualized, or misinterpreted, potentially delaying help-seeking. Awareness strategies should prioritize strengthening visible and culturally meaningful narratives about cancer within communities. This positions THPs as potentially important communicative actors capable of translating biomedical concepts into culturally recognizable forms. As a common first point of contact, THPs can serve as community health educators who help to demystify cancer and encourage early help-seeking among individuals who are reluctant to approach formal health services [16].

Gendered patterns in healthcare-seeking behavior were also evident, with men being less likely to attend clinics routinely and more likely to consult THPs for symptoms such as urinary difficulties that may indicate prostate cancer. This pattern was linked to perceptions that clinics are unfriendly toward men and the social norms associated with masculinity and healthcare avoidance [29]. Hence, THPs may occupy strategically important positions within gendered help-seeking pathways. However, this does not imply that THPs should function as extensions of the formal health sector or bear the responsibility for delayed care. Rather, the findings point to a dual approach that improves the responsiveness of biomedical services to men while recognizing the existing role of THPs in some care-seeking pathways.

Similar to the previous research by Mwaka et al. [13] and Msoka et al. [30], THPs expressed a willingness to contribute to cancer awareness and early detection. Their suggestions included training workshops, cancer awareness materials, access to clinical environments, collaboration with cancer survivors, structured community dialogues, and referral pathways with follow-up mechanisms. The findings suggest that THPs’ willingness to participate in cancer awareness initiatives is shaped not only by motivation but also by the extent to which institutional systems create meaningful opportunities for participation. This shifts attention away from solely individual knowledge deficits and toward the broader questions of inclusion, legitimacy, and infrastructural support.

Concerns regarding exploitation of intellectual property reflect broader debates regarding the protection of indigenous knowledge systems (IKS) [31] and challenge the assumption that motivation in the context of THPs in South Africa could lead to action [32]. They also highlight the need for sustained commitment by the National Department of Health to finalize IKS policies that ensure recognition, trust, and protection of IKS, thereby enabling THPs to engage in dialogue and collaboration with BHPs. Drawing on frameworks for IKS protection or examples of successful benefit-sharing agreements in other sectors could provide useful context for navigating these ethical and legal complexities [33].

Legitimacy has emerged as a key theme in both policy and implementation. Although national policy recognizes the potential role of THPs in prevention and early detection [9], policy recognition alone appears insufficient to support meaningful collaboration in practice, as institutional hierarchies and interpersonal mistrust continue to shape interactions between THPs and biomedical providers. This disconnect reflects a broader challenge in pluralistic health systems where policy commitments do not consistently translate into interprofessional collaboration, partly because of the evolving regulatory environment governing THPs in South Africa [34]. Power imbalances, absent referral pathways, and limited professional interactions are barriers to cooperation, as highlighted in Nkosi and Sibiya [18].

Within the COM-B model, these experiences illustrate how institutional conditions shape action opportunities. The THPs described challenges such as exclusion from consultations and dismissive attitudes from healthcare workers. The absence of recognized referral tools, communication channels, and formal contact points limits THPs ability to contribute, reinforcing hierarchies that marginalize them within care pathways. Addressing these challenges requires translating policy commitments into operational mechanisms, such as formally structured dialogue platforms and referral systems in addition to clearer guidance on professional roles and established boundaries [16,35].

Diversity within the traditional healing sector has emerged as a key factor shaping prospects for collaboration with biomedical providers. Variations in training pathways, experience, organizational affiliation, and governance structures influence how THPs understand their roles and engage with the formal health system. Some participants raised concerns about unregulated practitioners and the lack of unity within the profession, underscoring the sector’s heterogeneity and the challenges of establishing structured collaboration [36,37]. This diversity cautions against the assumption that THPs are uniformly prepared to participate in cancer-control initiatives [18,38]. Instead, collaboration should be strategic, initially focusing on areas aligned with existing practices, such as symptom awareness, myth reduction, timely referral, psychosocial support, and patient navigation, thus allowing partnerships to develop incrementally while maintaining patient safety.

### Strengths and limitations

This study has several strengths and limitations. A key strength is the inclusion of diverse THPs with varying training pathways, healing practices, spiritual orientations, organizational affiliations, and engagement with the formal health system, enabling a range of perspectives. However, recruitment through snowball sampling and professional networks may have overrepresented more connected practitioners, underrepresenting isolated THPs. The diversity of the THP7 sector also limits the generalizability of the findings across all THPs and settings. Nevertheless, the two-phase interview process, including member checking and summary validation, strengthened credibility and participant engagement. The study also did not explicitly examine how economic factors, livelihood dependence, may influence collaboration or referral practices.

## Conclusion

Our findings demonstrate that the involvement of THPs in cancer awareness and early detection is shaped by interconnected individual, community, and institutional factors. The THPs expressed willingness to support cancer awareness and referral but identified limitations in formal cancer training and uncertainty in managing cancer-related symptoms. At the community level, the THPs occupied trusted and culturally resonant roles that positioned them as important intermediaries in local help-seeking pathways, particularly in contexts shaped by stigma, fear, and gendered healthcare access. Institutionally, their involvement was constrained by limited recognition within the formal health system, weak referral pathways, and ongoing tensions between the traditional and the biomedical sectors. Despite these challenges, the THPs appeared strategically positioned to support culturally responsive cancer awareness and referral efforts within underserved communities. Further research incorporating patients, and the perspectives of BHPs, as well as the economic dimensions of collaboration, is needed to understand cancer-care pathways within pluralistic health systems better.

## Supplementary Material

Supplementary_file_Interview_guides_clean.docx

COREQ_Checklist_THP study_May.pdf

## Data Availability

The data that support the findings of this study are available upon request from the corresponding author, NAJ. The data are not publicly available because they contain information that can compromise the privacy of the research participants.
