# Supplementary material for: Bridging gaps in community cancer care: exploring Traditional Health Practitioners’ perceptions of common adult cancer and their role in raising community awareness and early detection in Soweto, South Africa
Source: Glob Health Action. 2026 Jul 13;19(1):2685414. doi: 10.1080/16549716.2026.2685414 (PMC13366635; doi:10.1080/16549716.2026.2685414)
Supplement: Supplementary_file_Interview_guides_clean.docx [file ZGHA_A_2685414_SM5362.docx]

**Appendix 1:** Traditional health practitioners’ IDI guide *(PHASE 1)*

Interview date:

Interviewer:

THP type (*Inyanga/Isangoma):* PID (e.g. THP001):

# Opening questions

1. Please tell me about your healing training journey (Probe: years as a healer, form of training received, registration status)
2. Kindly tell me about your experience working as a THP in Soweto.
3. Describe the type of clients you see on a day-to-day basis (Probe: ailments THPs commonly treat)
4. Do you encounter patients with cancer in your practice, if so, how often?
5. Could you kindly share if you have had a family member with cancer?

# Knowledge, perspectives and attitudes of THPs about cancer

1. What is your general understanding of cancer? (Probe: words that are used to describe cancer)
2. What do you think causes cancer? (Probe: for the types of cancer that THP recognises and treats)
3. What are the common symptoms of cancer according to you?
4. Kindly share some of the popular believes that this community holds about cancer (it’s causes, treatment and prognosis). Do you share the same beliefs? Why/why not?
5. Do you believe cancer can be prevented, and if so how?
6. Do you think there is stigma associated with cancer in the community? How does this stigma play out?
7. What is your general perspective about THPs management of cancer among SA communities?
8. Where do you think community members with cancer symptoms go for healing? Are such health facilities accessible to them?
9. What healthcare services, community awareness opportunities that are available for community members and stakeholders like THPs wanting to learn about cancer?

# THP’s current involvement with cancer care and community’s current resources

1. How do you diagnose cancer in your patients?
2. What traditional treatment and remedies do you use for patients with cancer?

17a. How do you approach such cases. Please take me though this journey from the time the client consults with you, the discussions, approaches to diagnoses, involvement of family, and/or medication dispensed, or ceremonies performed

17b. What role to THPs play in supporting cancer patients and their families, emotionally and spiritually?

**Appendix 2:** Traditional health practitioners’ IDI guide *(PHASE 2)*

Interview date:

Interviewer:

THP type (*Inyanga/Isangoma):* PID used in phase 1 (e.g. THP001):

# Opening statement

*We learnt a lot about your perspectives on cancer in our last interview. In this second interview, I would like us to start by briefly summarising these views and clarifying any questions I have from the last interview.*

1. From the last time we spoke, have you had additional thoughts about cancer or anything from our previous discussion?
2. Have you had a case or heard of a case involving a client with cancer since we last spoke? If yes, please tell me a little bit about this case.

# Knowledge of and experiences of THPs on breast cancer

17 (Reading of Vignette). I am going to read two short stories, and I would like your thoughts on these scenarios, specifically how would you diagnose, and advice this client about steps they should take? (Probe for medication dispensed, or ceremonies THP would perform)

Vignette sample

1. Nonceba is a 35-year-old woman. She complains about pains in her left breast. She has not visited the clinic, because she believes it has to do with her changing hormones, due to childbirth. She comes to you for assistance.
2. Bab’ Kgotso is a 50 year-old man. He comes to you stating that he has trouble urinating, and when he does, he notices blood on his urine.

# THP’s potential role in raising community awareness of breast and prostate cancer

(I am now interested in the potential role THPs can play in raising community awareness of breast cancer and prostate? In short, community awareness is the process of making people in the community aware of issues that affect them. Community awareness can be achieved through community dialogues such as *imbizo*, educational campaigns on print media or radio)

1. Have you been involved in raising community awareness about any disease outbreak in this community? How about breast or prostate cancer in your community? If yes, please tell me more about that.
2. Do you think THPs have or should have a role to play in raising community awareness about breast and prostate cancer? Why or why not? (Probe: what role could THPs play in

raising awareness and understanding of breast and prostate cancer symptoms in the community)?

1. In your opinion, what needs to be in place to allow for your involvement in community awareness of breast, prostate and other cancers? (Probe: Training needs, DoH support, Policies etc)
2. What do you think is the best approach to activate community participation for demanding preventative health services in Soweto?

# THPs referral approaches and education needs for cancer

*Referrals*

1. You mentioned referring clients to the clinic at various points in your practice. Tell me more about this. (Probe for: Facility, ways of referral, such as written letter, walking clients in)
2. How has this experience been? (Probe for: Wins, challenges, key lessons, role played by clients).
3. What is your perspective on referring cancer patients to Western healers (Probe for: What works, what should be improved)
4. How could THPs visibility be improved in the referral process. (Probe for: Ways to improve the process logistically, bi-directional communication and co-management of client)

*Education (reflect on previous trainings)*

1. How was the previous training delivered (Probe for: What worked work well, did not work well
2. What needs to be in place to ensure the success of the training (Probe for: Training resources, people to be involved, the role of the associations in delivering and future accountability system).
